# Bridging Data Gaps in Emergency Care: The NIGHTINGALE Project and the Future of AI in Mass Casualty Management

**DOI:** 10.2196/67318

**Published:** 2025-04-10

**Authors:** The NIGHTINGALE Consortium, Marta Caviglia

**Affiliations:** 1 Institute of Communication and Computer Systems Zografos Greece; 2 Scienze Mediche e Chirurgiche Università Cattolica del Sacro Cuore Rome Italy; 3 Disaster and Military Surgery Section ESTES European Society for Trauma and Emergency Surgery Milan Italy; 4 National Emergency Medical, Disaster, Ambulance and Blood Bank Service Magen David Adom Ashkelon, Israel Israel; 5 PARTICLE Lisbon Portugal; 6 INOV Instituto de Engenharia de Sistemas e Computadores Inovação Lisbon Portugal; 7 Carr Communications Dublin Ireland; 8 Netcompany-Intrasoft Luxembourg Luxembourg; 9 CESTEL Centro Español de Servicios Telemáticos S.A. Madrid Spain; 10 See Acknowledgments Novara Italy; 11 Center for Research and Training in Disaster Medicine, Humanitarian Aid and Global Health (CRIMEDIM) Università del Piemonte Orientale Novara Italy

**Keywords:** AI, technology, mass casualty incident, incident management, artificial intelligence, emergency care, MCI, data gaps, tool

## Abstract

In the context of mass casualty incident (MCI) management, artificial intelligence (AI) represents a promising future, offering potential improvements in processes such as triage, decision support, and resource optimization. However, the effectiveness of AI is heavily reliant on the availability of quality data. Currently, MCI data are scarce and difficult to obtain, as critical information regarding patient demographics, vital signs, and treatment responses is often missing or incomplete, particularly in the prehospital setting. Although the NIGHTINGALE (Novel Integrated Toolkit for Enhanced Pre-Hospital Life Support and Triage in Challenging and Large Emergencies) project is actively addressing these challenges by developing a comprehensive toolkit designed to support first responders and enhance data collection during MCIs, significant work remains to ensure the tools are fully operational and can effectively integrate continuous monitoring and data management. To further advance these efforts, we provide a series of recommendation, advocating for increased European Union funding to facilitate the generation of diverse and high-quality datasets essential for training AI models, including the application of transfer learning and the development of tools supporting data collection during MCIs, while fostering continuous collaboration between end users and technical developers. By securing these resources, we can enhance the efficiency and adaptability of AI applications in emergency care, bridging the current data gaps and ultimately improving outcomes during critical situations.

## Introduction: Challenges in AI Development for Prehospital Emergency Responses

In an era of rapid technological progress, artificial intelligence (AI) is significantly influencing health care practices. Its expanding applications currently encompass diagnostic support through medical image analysis, predictive modeling, personalized treatments, procedural assistance, and remote monitoring [1,2]. Beyond these routine uses, there is a demand driven by various initiatives—particularly in Europe—to develop AI-driven tools specifically for emergency contexts [3]. Emergency medicine, defined by its complex organizational requirements and the need for rapid, precise decision-making, presents significant opportunities for innovation, and integrating AI technology could enhance patient prioritization, decision support, workflow efficiency, and early warning systems [4,5]. Although AI-driven tools have already shown promise in improving diagnosis, triage, and decision-making in emergency department settings [4], in the context of prehospital emergency response, most research has remained at the proof-of-concept stage, highlighting the need for prospective validation to support real-world implementation [6]. Additionally, while integrating AI into prehospital emergency care offers the potential to augment responders’ performance, it also introduces the challenge of model training, which depends on accurate, relevant, and diverse datasets. Model training involves extensive data collection, processing, validation, and testing in the prehospital realm—steps that are crucial to enable AI models to identify patterns, minimize errors, and generalize across different scenarios [7]. Unfortunately, it is widely acknowledged that accurate data collection and analysis in prehospital settings, along with prospective research more generally, face a range of challenges and constraints [8]. The issue becomes particularly pronounced during prehospital response to mass casualty incidents (MCIs), where resources are extremely stretched and focused on saving lives, leaving minimal capacity for systematic and comprehensive data collection and management [9]. The defining characteristic of MCIs is their sudden and unpredictable nature, which, combined with the overwhelming number of casualties that exceed available resources, places significant strain on and often overwhelms local response systems [10,11]. In this context, essential traceable information, such as patient demographics, medical history, vital signs, and responses to emergency treatments, is often sparse, incomplete, and undocumented. Of note, even when continuous monitoring occurs in the prehospital environment (eg, in mobile intensive care units), the data are typically not recorded or stored, as they are deemed unnecessary for immediate treatment decisions. The absence of such data, curated accordingly, hinders the ability to create accurate models and limits the potential for AI to improve emergency responses and outcomes. Additionally, addressing data privacy and security concerns remains imperative, particularly to safeguard sensitive health information while ensuring regulatory compliance and interoperability, as clearly defined within the European General Data Protection Regulation (GDPR) framework [12]. Adherence to such regulation includes using secure methods for data storage, transmission, and processing to prevent unauthorized access or data breaches, while committing to equitable access, which ensures that the deployment of AI technologies does not exacerbate existing health care disparities [13]. Moreover, ethical frameworks should mandate explainability and transparency in AI algorithms and their decision-making processes, while balancing technological advancements with fundamental principles of patient autonomy, justice, and beneficence [3]. This approach fosters trust, ensures accountability, and aligns with the principles outlined in the Artificial Intelligence Act adopted by the European Parliament in March 2024 [14]. All these challenges were encountered by the Horizon 2020 European Union–funded NIGHTINGALE (Novel Integrated Toolkit for Enhanced Pre-Hospital Life Support and Triage in Challenging and Large Emergencies) project as it pursued its objective of developing a toolkit for supporting first responders in managing MCIs [15]. Building on existing knowledge, this viewpoint paper draws on insights from the NIGHTINGALE project to offer recommendations for enhancing the role of AI in supporting MCI responses in prehospital settings, in the attempt to bridge the current data gaps.

## Insights from the NIGHTINGALE Project

The NIGHTINGALE toolkit includes a wide range of tools, services, and applications designed to enhance triage during MCIs, optimize the use of resources, and facilitate coordinated responses among different stakeholders [15,16]. Among these, several AI tools and machine learning systems have been integrated to support decision-making and provide predictive insights, ultimately aiming to boost prehospital response efficiency during MCIs. These AI-driven tools are designed to assist in various aspects of emergency response, from real-time triage to resource allocation and casualty prognosis. However, their development within the NIGHTINGALE project experienced significant challenges, primarily due to the scarcity of prehospital MCI patient data to train AI models and the difficult compliance with the GDPR when these data were present. This shortage forced engineers to rely on in-hospital datasets [17], which, while useful, do not accurately reflect the conditions of casualties in prehospital settings or capture their progress after field interventions. Indeed, this data issue extends beyond mere quantity. Equally important is the quality of the data, which is essential for ensuring accurate and effective training of AI-driven applications, thereby preventing the “garbage in, garbage out” phenomenon that might arise [18]. For instance, in cases where systems fail to align patient data with treatment details, such as whether a patient is receiving oxygen or undergoing fluid resuscitation, the accuracy of AI predictions can be compromised. Trauma cases add complexity, as rare but critical scenarios (like severe abdominal injuries or pregnant women with high intracranial pressure) require AI to draw from a vast and diverse data pool to learn the varied patterns and associated responses. Additionally, AI must account for trauma mechanisms, care quality in the field, and treatment timelines; thus, comprehensive data on field diagnoses, emergency department outcomes, and treatment results are essential. Consequently, data curation, preprocessing, and filtering are critical data collection steps, allowing AI models to function effectively in a wide range of clinical situations. To address these challenges, the NIGHTINGALE project has focused on integrating continuous monitoring of the vital signs of casualties within a comprehensive set of tools, designed also to record demographic information, patient details, and administered treatments [15]. One significant advancement is the implementation of integrated sensors and applications, including wearable devices, to continuously monitor and transmit vital signs to a central storage medium, along with a system that facilitates data interoperability, enabling seamless data access, querying, and retrieval, regardless of the data collection source [15]. Indeed, when fusing data from various sensors and applications, it becomes essential to manage and represent the data in a standardized manner. Independently of the data’s origins—whether from wearable devices, medical records, or other sources, standardization ensures that it can be accessed and used by AI-driven applications without concerns about format or structure. This approach allows these systems to remain agnostic to specific collection methods or data structures, ensuring seamless interoperability and enabling efficient integration and utilization of diverse datasets for decision-making [19]. Though these innovations point in a promising direction by offering valuable data during MCIs, significant research and development are still required to make them fully operational. Further refinement and validation (including clinical validation) are essential to ensure the devices can effectively support first responders and reliably collect crucial data, without adding complexity or delaying patient care. Moreover, their integration should be gradual to avoid disrupting established workflows; instead, they should progressively enhanced, which may require the adaptation of current operational protocols. In conclusion, continued research is vital to develop reliable tools that offer tangible benefits for improving prehospital MCI responses and to generate high-quality, accurate data necessary for training AI-driven systems—an effort that, due to the rarity of MCIs, may require considerable time.

## Toward Scalable AI Solutions in MCI Management

Acknowledging that the integration of technology, and especially AI tools into MCI management, remains in its infancy, with few real-world case studies available to demonstrate the effectiveness of these tools in high-stakes, real-time scenarios, we present some recommendations to bridge the current data gaps and support the integration of such technology in prehospital MCI management.

### Recommendation 1: Leveraging Transfer Learning

To address the limitations posed by data scarcity, researchers are increasingly exploring transfer learning as a practical and effective solution [20,21]. This approach leverages large pretrained models that can be adapted and fine-tuned using smaller, domain-specific datasets [20,21]. By doing so, it becomes possible to develop reliable AI systems without necessitating the collection of exceptionally large datasets, which is particularly challenging in emergency care and MCI settings, where data acquisition is often constrained. Recognizing the potential of transfer learning to overcome the impracticalities of building a vast and diverse dataset from scratch, the NIGHTINGALE Consortium advocates for a coordinated, large-scale research initiative at the European Union level. Such a project would aim to facilitate the collection of diverse and extensive data across multiple institutions and to create a robust pretrained AI model. This model could then be tailored by individual health care organizations to address their unique operational and clinical requirements, fostering the integration of advanced AI tools in prehospital emergency care and improving outcomes in MCI management.

### Recommendation 2: Enhancing Prehospital Data Collection Mechanisms

To bridge the critical gap in prehospital data availability, the development and implementation of robust mechanisms for systematic data collection during MCIs should be prioritized. These mechanisms should integrate advanced, user-friendly technologies that enable first responders to capture essential patient and situational data without compromising their primary focus on saving lives. Sensor-based systems capable of continuous monitoring and documentation could mitigate the manual burden on responders and ensure comprehensive data capture in high-stress environments. On the operator side, reliable speech-to-text tools could facilitate the recording of crucial treatment and life-saving procedures without disrupting first responders’ actions. Fostering the use of real-time data collection tools and the implementation of standardized protocols will contribute to the gradual development of datasets that could both support the fine-tuning of pretrained AI models and enable accurate after-action reviews and performance debriefings [22].

### Recommendation 3: Building Strategic Partnership

The NIGHTINGALE Consortium emphasizes the importance of establishing strategic partnerships to ensure the sustainability and scalability of its initiatives, particularly as current funding frameworks are limited in scope and duration. Indeed, the development of advanced technologies for emergency response requires the ongoing and long-term involvement of end users and technicians, which cannot be confined to a 3- or 4-year partnership. Instead, projects with such ambition should be automatically funded until a technology readiness level is achieved that convinces industry to purchase and further implement the technology. By fostering continuous collaborations with diverse stakeholders, including health care institutions, research entities, industry leaders, and policy makers, these partnerships can create a robust support network that drives innovation and addresses operational challenges. Despite the intrinsic limitations of a 3-year duration, the NIGHTINGALE project served as a compelling example of the effectiveness of this approach, as the close collaboration between technological developers and end-user partners was instrumental in achieving a user-centered approach to technological development. End-user partners provided critical and evidence-based insights that shaped the design and implementation of tools to meet real-world needs effectively [23,24]. In addition to these aspects, strategic alliances can facilitate access to larger and more diverse datasets, while promoting the development of standardized protocols for data sharing and interoperability. Moreover, such collaborations can enhance the efficiency and adaptability of AI models by pooling expertise and resources, ensuring that AI-driven solutions are not only effective but also flexible enough to meet the evolving demands of emergency care environments.

## Abbreviations

AI: artificial intelligence
GDPR: General Data Protection Regulation
MCI: mass casualty incident
NIGHTINGALE: Novel Integrated Toolkit for Enhanced Pre-Hospital Life Support and Triage in Challenging and Large Emergencies
